# *Cxcl9*-deficiency attenuates the progression of post-traumatic osteoarthritis in mice

**DOI:** 10.1007/s00011-025-02013-8

**Published:** 2025-03-06

**Authors:** Antonia Donat, Weixin Xie, Shan Jiang, Laura Janina Brylka, Thorsten Schinke, Tim Rolvien, Karl-Heinz Frosch, Anke Baranowsky, Johannes Keller

**Affiliations:** 1 Department of Trauma and Orthopedic Surgery, University Medical Center Hamburg-Eppendorf, Martinistraße 52, 20251 Hamburg, Germany; 2 Department of Osteology and Biomechanics, University Medical Center Hamburg-Eppendorf, 20251 Hamburg, Germany; 3 Department of Trauma Surgery, Orthopedics and Sports Traumatology, BG Hospital Hamburg, 21033 Hamburg, Germany

**Keywords:** CXCL9, Osteoarthritis, ACLT, Subchondral bone, Cartilage, Osteophytes, Synovitis

## Abstract

**Objective:**

Osteoarthritis (OA) is one of the leading causes of disability in the aging population. While about 10% of the adult population is affected by OA, there is to date no curative treatment and joint replacement surgery remains the only option for treating end-stage OA. Previous studies found elevated levels of the chemokine C-X-C motif ligand 9 (CXCL9) in the synovial fluid of OA knees. However, the exact role of CXCL9 in OA progression is still unknown.

**Methods:**

Female wild-type and *Cxcl9*-deficient mice were challenged with a unilateral anterior cruciate ligament transection (ACLT). Joint destruction in early and late stages of experimental OA was assessed using micro-CT scanning, histological scoring, histomorphometry, and gene expression analysis.

**Results:**

Inactivation of *Cxcl9* protected from cartilage destruction and osteophyte formation in post-traumatic OA in mice. Similarly, indices of joint inflammation including synovitis and expression of pro-inflammatory interleukin-1beta were reduced in OA knees of *Cxcl9*-deficient mice. However, bone erosion and pathophysiological changes in the subchondral bone compartment remained unaffected in *Cxcl9*-deficient mice with experimental OA.

**Conclusion:**

Our results point towards a pro-inflammatory role of CXCL9 in OA and identify a potential new target for the pharmacological treatment of OA.

**Supplementary Information:**

The online version contains supplementary material available at 10.1007/s00011-025-02013-8.

## Introduction

As a result of injury, aging, abnormal weight bearing or hereditary conditions, articular cartilage can degenerate and lead to joint destruction, commonly referred to as osteoarthritis (OA). This chronic condition is one of the leading causes of disability and one of the most common pathologies relevant to orthopedic surgery, affecting over 500 million people worldwide [1, 2]. Due to the demographic change and increasing age of the population, even more OA patients and hence joint replacement procedures are to be expected over the next decades [3]. OA most commonly affects weight-bearing joints like the hip and knee joint. Pathophysiological changes seen in OA include a decreased joint space, cartilage defects, abnormal remodeling of the subchondral bone, and the formation of osteophytes [4, 5]. Clinically, OA most commonly leads to pain and stiffness of the affected joints and can result in severe disability. In early stages, lifestyle interventions and NSAID treatment are recommended to reduce clinical symptoms, even though efficacy of the latter is still lacking profound evidence [6]. More importantly, these measures do not affect disease progression and currently there is no cure to attenuate or stop the degenerative process. Therefore, extended joint replacement surgeries, known as arthroplasty, represent the only treatment option for advanced OA. These procedures yield multiple risks, such as periprosthetic joint infection, aseptic loosening or persistent pain, which often require one or multiple revision surgeries. In addition to these complications, the limited implant survival time highlights that preventive OA therapies are of utmost importance to improve patient care.

The C-X-C motif ligand (CXCL) chemokine family is known for its regulatory role in inflammatory processes and the immune system. In this regard, CXCL9 and its corresponding receptor, CXC motif chemokine receptor 3 (CXCR3), have previously been investigated in oncological research and various other medical fields. Here, CXCL9 was shown to act as a type 1 helper T cell (Th1) chemoattractant, which plays an important role in the growth, activation and movement of cells associated with immune and inflammatory responses [7]. It has also been implicated in tumor growth inhibition and angiogenesis [8, 9].

In bone, CXCL9 has been reported to affect both bone formation and bone resorption. Osteoblast-derived CXCL9 was shown to inhibit bone angiogenesis and thus impair bone formation. In addition, CXCL9 has been demonstrated to regulate recruitment of osteoclast precursors to the bone matrix in fish, thereby modulating bone resorption [10]. In addition, neutralizing CXCL9 in experimental osteoporosis prevented bone loss in mice by increasing bone formation as well as decreasing bone resorption [11]. Clinically, increased CXCL9 blood levels were reported to be associated with an increased risk for hip fracture in men but not in women [12]. Regarding orthopedic surgery, several clinical studies have reported elevated CXCL9 levels in OA patients. For example, in a study of 34 OA patients undergoing unicompartmental or bicompartmental knee arthroplasty, synovial CXCL9 levels were associated with worse preoperative knee function [13]. In addition, Yang et al. showed that CXCL9 levels were elevated in the synovial fluid and serum of OA patients [14].

Based on its pleiotropic function in the immune system and in bone remodeling, alterations of which also play a critical role in the progression of OA, CXCL9 may thus be of critical importance in the pathogenesis of OA. Therefore, this study was designed to investigate the effect of *Cxcl9* ablation on the progression of experimental OA.

## Materials and methods

### Animal experiments

Ethical approval for all experiments was obtained prior to project start by the local legal representative animal rights protection authorities (Behörde für Justiz und Verbraucherschutz Hamburg, N21/101). Female WT mice and *Cxcl9*-deficient mice (B6.129S4-*Cxcl9*^*tm1Jmf*^/J) with a C57BL/6 genetic background were used for the experiments. Homozygous *Cxcl9*-deficient mice display an increased risk for certain viral and bacterial infections. Nevertheless, they are viable, fertile and have a normal life expectancy. The mice were derived from Jackson Laboratory (JAX stock #030285) and originally generated by Park et al. [15]. During the course of the experiment, the mice were kept in a SPF facility under standardized conditions (12 h circadian rhythm, 22 °C room temperature, 55% humidity). They were housed with littermates in stable groups of three to four animals per cage and received a standard diet and water *ad libitum.*

### Materials

For animal experiments the following materials were used: Buprenorphine (Buprenovet^®^, Richter Pharma AG, Wels, Austria), Clindamycin (Hikma Pharma GmbH, Martinsried, Germany), Metamizole (Novaminsulfon-ratiopharm^®^, Ratiopharm, Ulm, Germany).

### Surgery

At the age of 12 weeks, the mice received a unilateral transection of the anterior cruciate ligament of the right hindlimb. Surgery was performed as previously described [16]. In brief, the mice received Clindamycin (150 mg·kg^− 1^) and Buprenorphine (0.1 mg·kg^− 1^) preoperatively and were anesthetized using a continuous flow of 1.5% isoflurane and 2 l·min^− 1^ oxygen. A longitudinal incision was made on the femur to expose the knee joint. After dissection of the vastus lateralis of the musculus quadriceps femoris, the patella was dislocated medially, and the anterior cruciate ligament was transected under direct visualization. Positive anterior tibial dislocation sign confirmed the successful transection of the respective ligament. Muscle and skin were closed using a simple interrupted suture. For postoperative pain-relief, Metamizole (1 mg·ml^− 1^) was given for three consecutive days via the drinking water. The mice were sacrificed 4 and 8 weeks post-operatively and samples collected subsequently.

### Sample preparation

After scarification, both knee joints were fixed in 10% formalin for 24 h without internal or external rotation. Afterwards, samples were scanned using micro-CT (µCT). Subsequently, the samples were decalcified in 0.5 M EDTA solution for 1 week at 4 °C under shaking conditions, dehydrated in 70–100% ethanol in an ascending order (2065 HISTOMASTER No. 20652401, RoWi Elektronik, Steffenberg, Germany) and embedded in paraffin.

### Micro-computed tomography

To assess structural bone parameters, operated and contralateral knee joints were scanned and reconstructed using µCT (VivaCT 80, SCANCO Medical AG, Brüttisellen, Switzerland) at voxel resolution of 15.6 μm, 400 ms integration time, 70 kVp and 113 µA. Subsequently, osteophyte volume and structural parameters of the tibial subchondral trabecular bone were evaluated as described elsewhere [17]. Assessed parameters included Bone Volume per Tissue Volume (BV/TV), Trabecular Number (Tb.N), Trabecular Thickness (Tb.Th) and Trabecular Separation (Tb.Sp). For evaluation of osteophyte volume, contours were drawn manually on slides that displayed additional bone formation around the knee joint with adherence to the distal femur or proximal tibial. A consistent threshold was used on each anatomical site.

### Histology

4 μm longitudinal sections of the knees were cut using a Microtec rotation microtome. For histological evaluation of osteophyte formation, bone erosion and OARSI score, Bone-Inflammation-Cartilage (BIC) staining was performed according to a modified protocol combining Van Gieson’s stain, Fast Green and Safranin O [18]. OARSI scoring was performed according to current recommendations [19]. In consideration of loss of Safranin-O and structural changes such as fibrillations, loss of cartilage, vertical clefts and the degree of affected surface lamina, a grade on a scale from 0 to 6 was determined. Using the same sections, the formation of osteophytes was assessed histologically. In this regard, osteophyte size and maturity were evaluated on a scale from 0 to 3 as described elsewhere [20]. Furthermore, the level of bone erosion on a scale from 0 to 3 was estimated as described by Jackson et al. [21]. For evaluation of synovitis according to Lewis et al. [22], H&E staining was performed using Mayer’s hemalum solution (Sigma Aldrich, Merck, Darmstadt, Germany) as previously described [23]. Subsequently, cell density and thickening of the synovial lining cell layer was scored on a scale from 0 to 3 and summed for all four quadrants of the knee. All four quadrants of the knee joint were scored separately, and the sum value calculated representatively for the whole knee joint. Each scoring was performed independently by two blinded investigators. To evaluate numeric parameters of osteoclasts in the subchondral bone, TRAP staining was performed [24, 25]. Osteoclasts were evaluated by counting TRAP-positive, multinucleated cells on the respective slides using the OsteoMeasure histomorphometry system (Osteometrics Inc., Atlanta, USA).

The thickness of hyaline cartilage, calcified cartilage, and subchondral bone plate was measured in H&E-stained sections as described previously [17]. Briefly, a 400 μm × 300 μm area in the center of medial tibial plateau was measured. The OsteoMeasure system was used for drawing the boundaries of hyaline cartilage, calcified cartilage, and subchondral bone plate, and calculating the thickness automatically. The parameters included the ratio of hyaline cartilage thickness to calcified cartilage thickness (HC.Th/CC.Th), percentage of hyaline cartilage area per total cartilage area (HC.Ar/T.Ar (%)), and subchondral bone plate thickness (SBP.Th (µm)).

### RNA extraction and qRT-PCR

Operated and contralateral knees were dissected 4 and 8 weeks post-operatively. Concomitantly, operated and contralateral knees of an independent WT group (*n* = 6 female mice, C57Bl/6J genetic background, 12–14 weeks old) were harvested 2, 4 and 8 weeks post-operatively (data exclusively displayed in Supplementary Fig. 1). Muscles and soft tissue were removed manually. Subsequently, the samples were snap-frozen in liquid nitrogen and stored in -80 °C until further processing. To extract the RNA, samples were homogenized in TRIzol (Sigma Aldrich, Merck, Darmstadt, Germany) using an Ultra Turrax (IKA Labortechnik, Staufen, Germany). For further processing, a standardized purification protocol employing a NucleoSpin RNA kit (Macherey-Nagel, Düren, Germany) was conducted. Using the NanoDrop 2000 system (NanoDrop Technology), concentration and quality of the isolated RNA was assessed. cDNA was derived through reverse transcription using a cDNA synthesis kit (ProtoScript First Strand cDNA Synthesis Kit, New England BioLabs, Ipswich, Massachusetts, USA). For gene expression analysis, quantitative real-time PCR of osteogenic, cartilage and inflammatory markers was carried out using TaqMan Assay-on-Demand primer sets (Applied Biosystems‎). The housekeeping gene Glyceraldehyde-3-phosphate dehydrogenase (*Gapdh*) was used as a reference for all groups. Relative quantification was performed using the ΔΔCT method.

### Statistical analysis

The group size of 8 animals per group was calculated according to the main outcome parameter cartilage destruction to obtain an alpha of 0.05 and power of 0.8 with an assumed mean difference of 72%. The researchers were blinded during operation, sample processing and data analyses. For evaluation of differences between WT and *Cxcl9*-deficient group in µCT and histomorphometry, unpaired two-tailed Students t-Test was used. For gene expression analysis, non-parametric Mann-Whitney-U-test was used. Unless stated otherwise, data is displayed as box plots with median value and minimum and maximum whiskers. Differences were considered significant at *P* < 0.05.

## Results

### Attenuation of post-traumatic OA in Cxcl9-deficient mice

To delineate a potential role of CXCL9 and its receptor CXCR3 in experimental OA, we first monitored gene expression in OA and sham-operated knees in 12-week-old WT mice subjected to anterior cruciate ligament transection (ACLT). Here, a gradual increase of *Cxcl9* expression in entire joints was observed during OA progression, reaching significance 8 weeks post-operatively (**Supplementary Fig. 1A**). The levels of *Cxcr3*, encoding the CXCL9 receptor, were elevated in a similar manner and were significantly increased after 4 weeks already (**Supplementary Fig. 1B**). As the severity of OA correlates with disease duration, these results pointed towards a potential pathophysiological role of *Cxcl9* in the progression of OA.

**Fig. 1:**
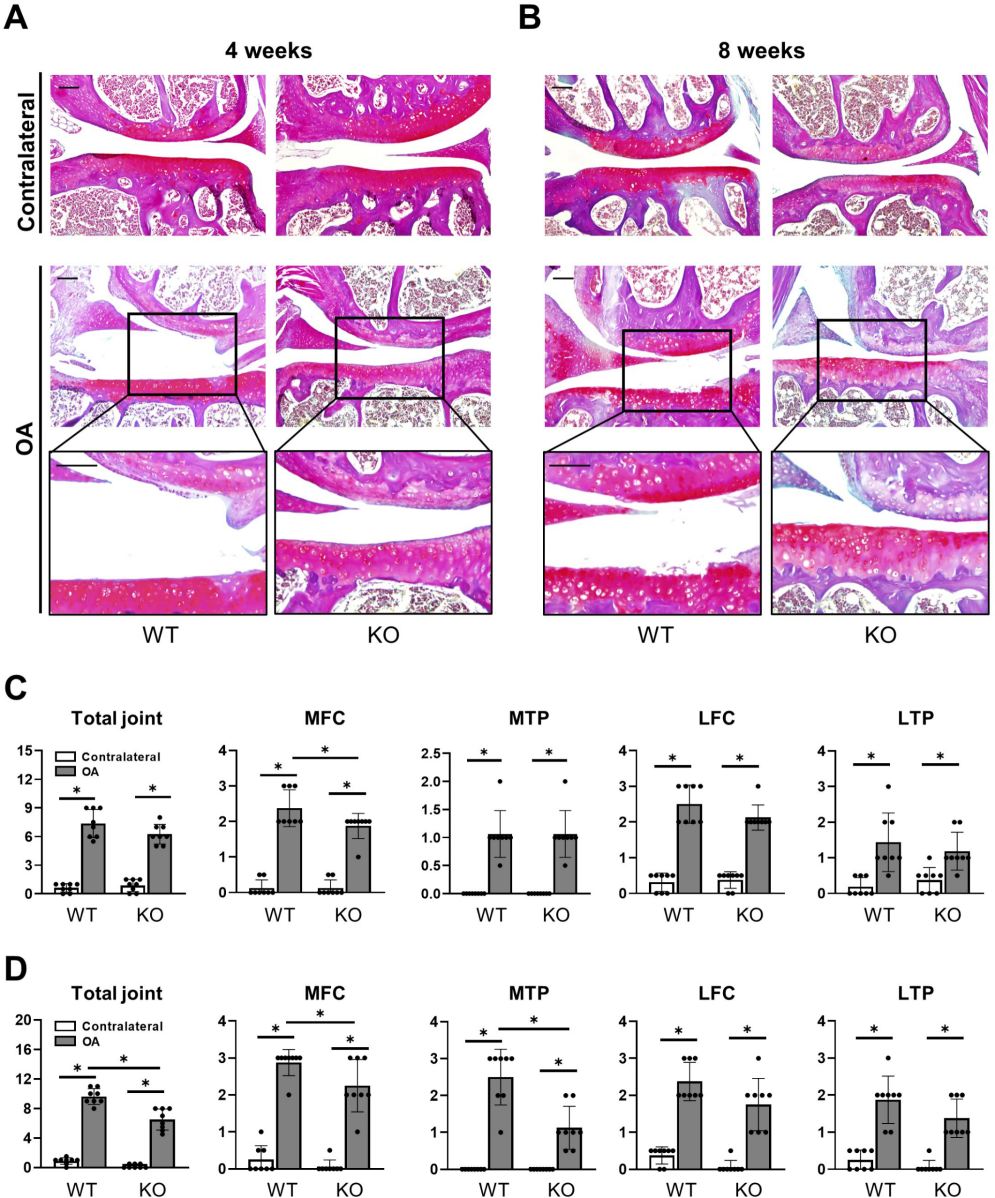
Cxcl9-deficiency alleviates cartilage degeneration after ACLT. **A** Representative BIC-stained images of medial compartments in contralateral and OA knees at 4 weeks after ACLT (scale bar = 100 μm). **B** Representative BIC images of medial compartments in contralateral and OA knees at 8 weeks after ACLT (scale bar = 100 μm). **C** Histological OARSI scoring of total joint, medial femoral condyle (MFC), medial tibial plateau (MTP), lateral femoral condyle (LFC), and lateral tibial plateau (LTP) at 4 weeks after ACLT. **D** Histological OARSI scoring of total joint, MFC, MTP, LFC, and LTP at 8 weeks after ACLT. The data are expressed as the means ± SD, *n* = 8 per group as indicated. Two-way ANOVA followed by Tukey’s post-hoc test was used for data analysis. ^*^P < 0.05 compared as denoted by bar

To further characterize the role of CXCL9 in OA, we next challenged WT and *Cxcl9*-deficient mice with ACLT to induce post-traumatic OA and analyzed the operated and contralateral knee joints at early (4 weeks) and late (8 weeks) timepoints. Post-traumatic OA developed in ACLT knees of both WT mice and *Cxcl9*-deficient mice, as indicated by cartilage erosion (Fig. 1A-B). However, an alleviated course of OA progression was observed in *Cxcl9*-deficient mice histologically. Scoring the pathological changes semi-quantitatively according to OARSI guidelines 4 weeks after ACLT, a reduced progression of OA with significant results in the medial femoral compartment (MFC) was observed in *Cxcl9*-deficient mice (Fig. 1C). After 8 weeks, significantly reduced joint destruction in *Cxcl9*-deficient mice was not only found in the MFC but also in the medial tibial compartment (MTP) and the sum of all four knee quadrants, indicating a protective effect of *Cxcl9* ablation on cartilage integrity during post-traumatic OA (Fig. 1D).

**Fig. 2:**
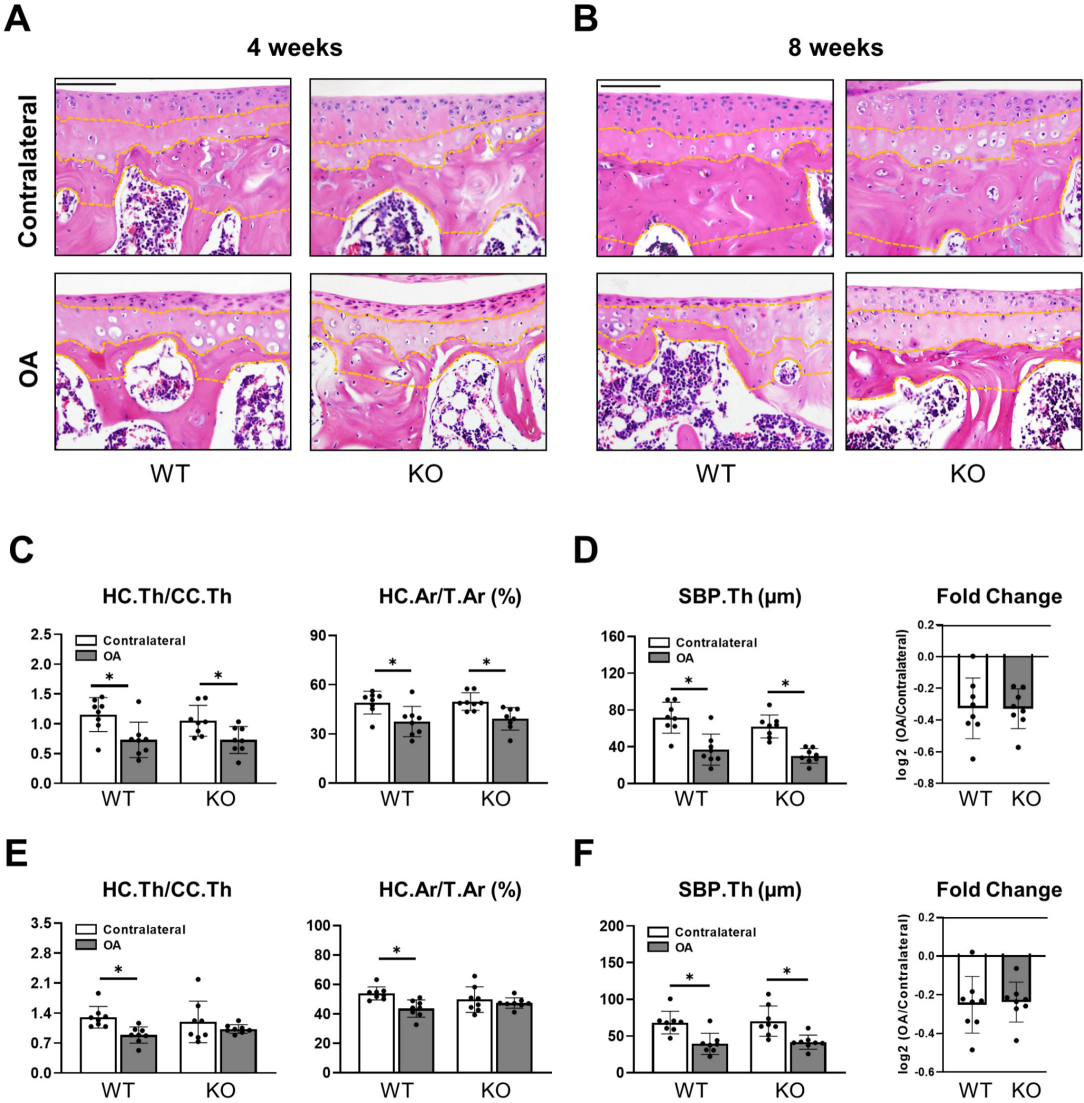
Cxcl9-deficiency prevents hyaline cartilage loss after ACLT. **A** Representative HE-stained images of medial tibial cartilage in contralateral and OA knees 4 weeks after ACLT (scale bar = 100 μm). **B** Representative HE-stained images of medial tibial cartilage in contralateral and OA knees 8 weeks after ACLT (scale bar = 100 μm). **C** Quantification of the ratio of hyaline cartilage thickness to calcified cartilage thickness (HC.Th/CC.Th) and percentage of hyaline cartilage area per total cartilage area (HC.Ar/T.Ar) 4 weeks after ACLT. **D** Quantification of subchondral bone plate thickness (SBP.Th) with comparisons of fold change (OA knee to contralateral knee) 4 weeks after ACLT. **E** Quantification of HC.Th/CC.Th and HC.Ar/T.Ar 8 weeks after ACLT. **F** Quantification of SBP.Th with comparisons of fold change (OA knee to contralateral knee) 8 weeks after ACLT. The data are expressed as the means ± SD, *n* = 8 per group as indicated. Two-way ANOVA followed by Tukey’s post-hoc test and two-tailed Student’s t-test were used for data analysis. ^*^P < 0.05 compared as denoted by bar

Assessing cartilage pathology in more detail, reduced cartilage integrity and loss of hyaline cartilage was observed in H&E-stained histology slides of the operated knees of both genotypes at early and late timepoints (Fig. 2A, B). Evaluation of the articular cartilage 4 weeks after ACLT revealed that the ratio of hyaline to calcified cartilage was not significantly different from the *Cxcl9*-deficient group (Fig. 2C). At 8 weeks post-operatively however, the ratio of hyaline to calcified cartilage in ACLT knees was equal to the contralateral control knees in *Cxcl9*-deficient mice (Fig. 2E), indicating that *Cxcl9* deficiency also protects from cartilage calcification in later stages of post-traumatic OA. Finally, histological evaluation of the subchondral bone plate thickness revealed a reduced diameter in OA knees compared to the contralateral control without any differences between *Cxcl9*-deficient and WT mice at both timepoints (Fig. 2D, F).

### OA-associated pathological changes in the subchondral bone are Cxcl9-independent

**Fig. 3:**
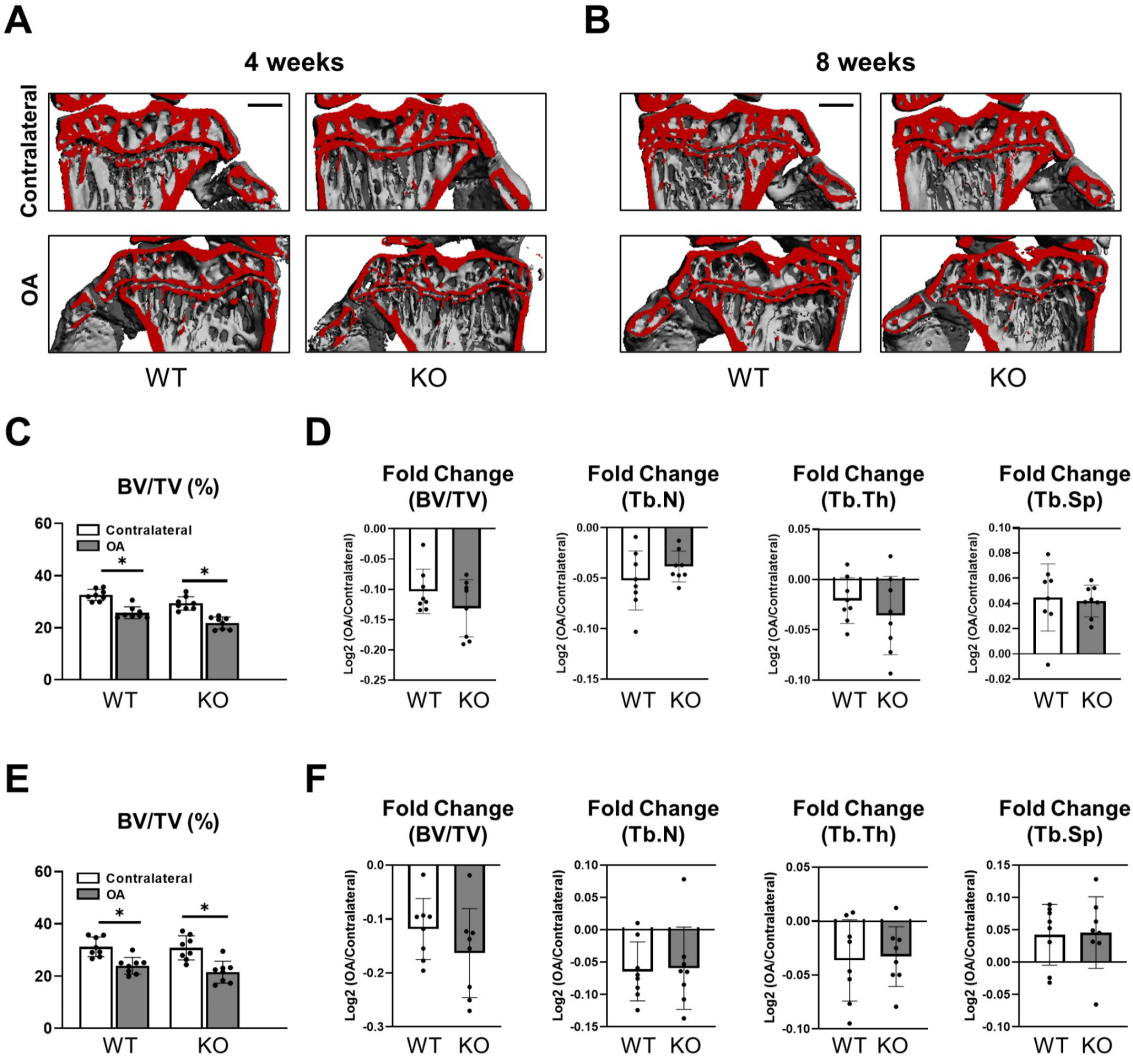
Cxcl9-deficiency does not affect subchondral bone loss after ACLT. **A** Representative µCT reconstruction images of tibial subchondral bone 4 weeks after ACLT (scale bar = 500 μm). **B** Representative µCT reconstruction images of the tibial subchondral bone 8 weeks after ACLT (scale bar = 500 μm). **C** Quantification of tibial subchondral bone for bone volume fraction (BV/TV) 4 weeks after ACLT. **D** Comparisons of fold change (OA knee to contralateral knee) for BV/TV, trabecular number (Tb.N), trabecular thickness (Tb.Th), and trabecular separation (Tb.Sp) 4 weeks after ACLT. **E** Quantification of tibial subchondral bone for BV/TV 8 weeks after ACLT. **F** Comparisons of fold change (OA knee to contralateral knee) for BV/TV, Tb.N, Tb.Th, and Tb.Sp 8 weeks after ACLT. The data are expressed as the means ± SD, *n* = 8 per group as indicated. Two-way ANOVA followed by Tukey’s post-hoc test and two-tailed Student’s t-test were used for data analysis. ^*^P < 0.05 compared as denoted by bar

Using micro-computed reconstruction images of ACLT and control knees, pathological changes in the tibial subchondral bone compartment were assessed in WT and *Cxcl9*-deficient mice. Here, no visible changes were observed macroscopically between the two groups at early and late-stage post-traumatic OA (Fig. 3A, B). Quantification of structural parameters in the subchondral trabecular bone of the tibia revealed bone loss in ACLT knees with no differences in bone volume per tissue volume between the mice of both genotypes 4 weeks post-operatively (Fig. 3C). The ratio of bone volume per tissue volume compared to the contralateral side as well as the fold change of trabecular parameters including trabecular numbers, thickness, and separation was also unaltered (Fig. 3D). Similarly, the changes in bone volume per tissue volume and trabecular parameters of the subchondral bone were similar in *Cxcl9*-deficient and WT mice in late-stage OA (Fig. 3E, F). Hence, *Cxcl9* does not affect subchondral bone remodeling in experimental OA.

### Bone resorption in subchondral bone is not affected by Cxcl9-deficiency

**Fig. 4:**
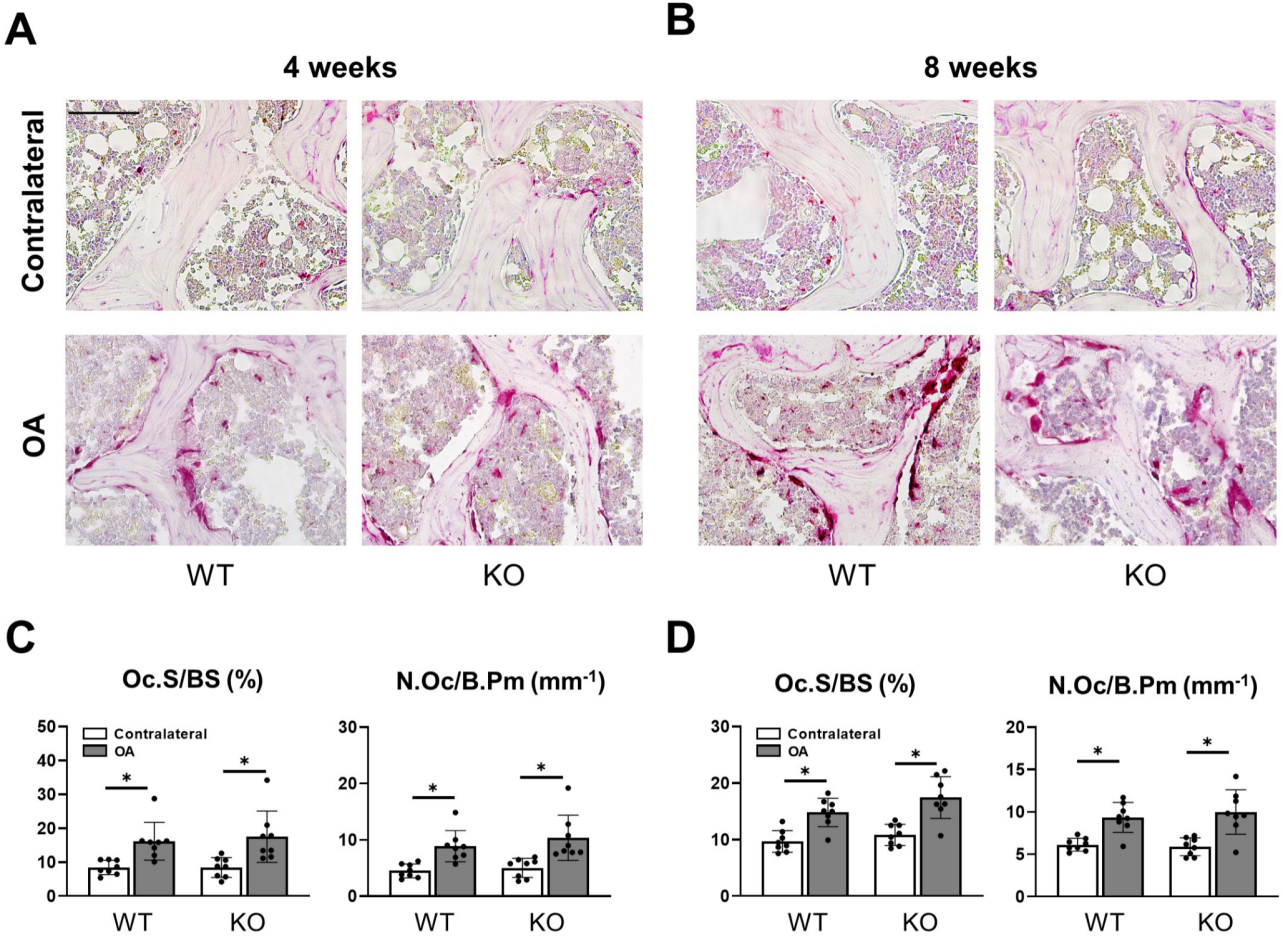
Cxcl9-deficiency does not affect bone resorption in subchondral bone loss after ACLT. **A** Representative Trap-stained images of tibial subchondral bone 4 weeks after ACLT (scale bar = 100 μm). **B** Representative Trap-stained images of tibial subchondral bone 8 weeks after ACLT (scale bar = 100 μm). **C** Quantification of osteoclast surface per bone surface (Oc.S/BS) and number of osteoclasts per bone perimeter (Oc.N/B.Pm) in tibial subchondral bone 4 weeks after ACLT. **D** Quantification of Oc.S/BS and Oc.N/B.Pm in tibial subchondral bone 8 weeks after ACLT. The data are expressed as the means ± SD, *n* = 8 per group as indicated. Two-way ANOVA followed by Tukey’s post-hoc test was used for data analysis. ^*^P < 0.05 compared as denoted by bar

To further characterize the loss of bone observed in the subchondral bone of OA knees, TRAP-activity staining of both knees of WT and *Cxcl9-*deficient mice was performed (Fig. 4A, B). Manual counting of osteoclast parameters 4 weeks after the operation revealed increased osteoclast surface and numbers in both groups (Fig. 4C). Assessment of ACLT and control knees 8 weeks post-operatively demonstrated comparable findings with an increase in osteoclast surface and numbers in both *Cxcl9*-deficient and WT mice (Fig. 4D). In sum, subchondral bone loss in experimental OA seems to be independent of CXCL9.

### Osteophyte formation is reduced in Cxcl9-deficient mice

**Fig. 5:**
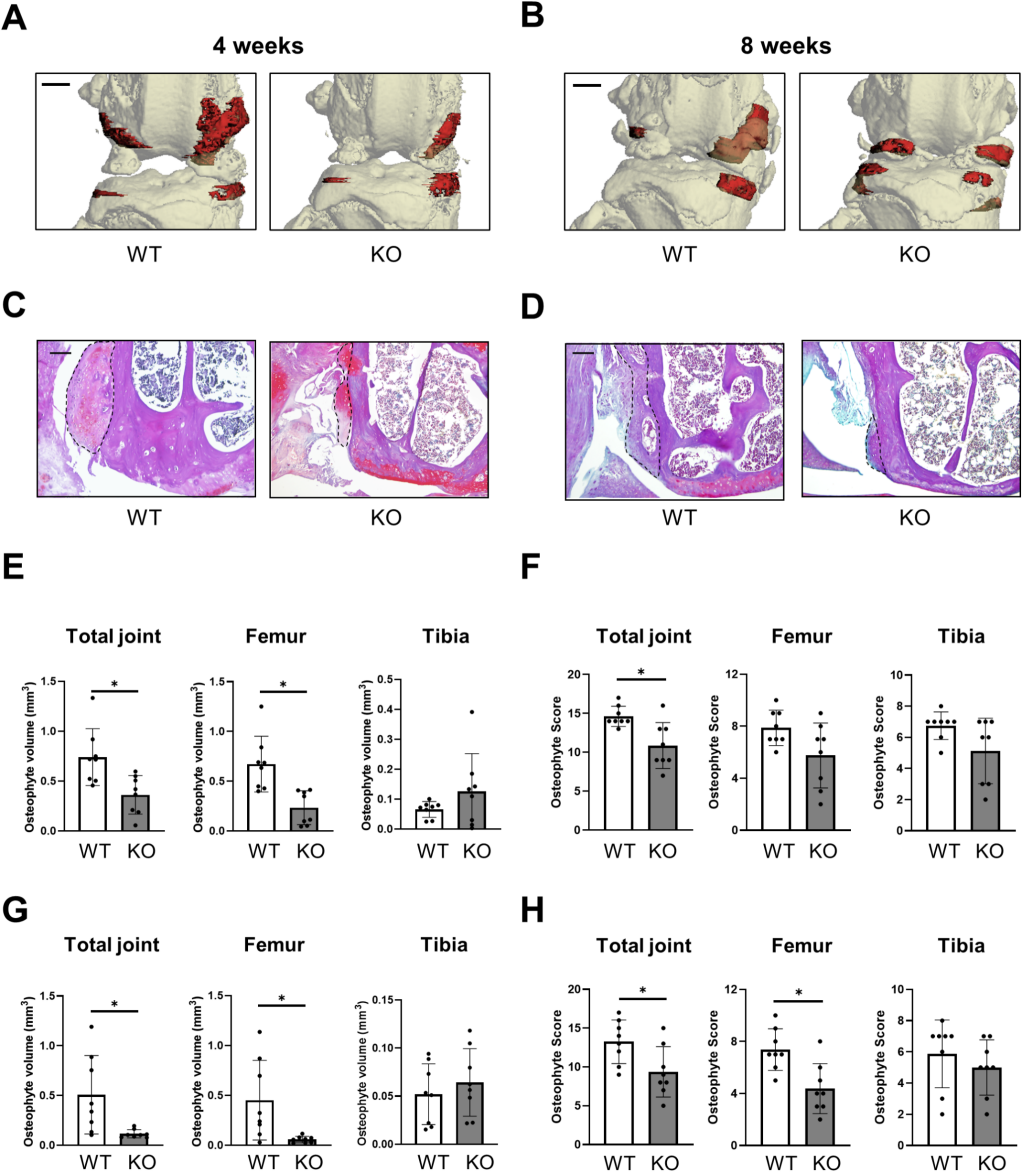
Cxcl9-deficiency inhibits osteophyte formation after ACLT. **A** Representative µCT reconstruction images of osteophytes 4 weeks after ACLT (scale bar = 500 μm). Osteophytes are shown in red. **B** Representative µCT reconstruction images of osteophytes 8 weeks after ACLT. **C** Representative BIC staining images of osteophytes 4 weeks after ACLT (scale bar = 100 μm). Osteophytes are marked by black dotted line. **D** Representative BIC staining images of osteophytes 8 weeks after ACLT. **E** Quantification of osteophyte volume through µCT evaluation in total joint, femoral side and tibial side 4 weeks after ACLT. **F** Histological osteophyte scoring of total joint, femoral side and tibial side 4 weeks after ACLT. **G** Quantification of osteophyte volume through µCT evaluation in total joint, femoral side and tibial side 8 weeks after ACLT. **H** Histological osteophyte scoring of total joint, femoral side and tibial side 8 weeks after ACLT. The data are expressed as the means ± SD, *n* = 8 per group as indicated. Two-tailed Student’s t-test was used for data analysis. ^*^P < 0.05 compared as denoted by bar

Using µCT and histological evaluation, pathological changes in ACLT knees, including osteophyte formation and joint deformity, were observed in both WT and *Cxcl9-*deficient mice at both early and late time points (Fig. 5A-D). However, macroscopically visible changes in *Cxcl9*-deficient mice were less aggravated at early and late stages of OA. At 4 weeks after ACLT, radiological quantification of the femoral compartment showed a significantly reduced osteophyte volume in *Cxcl9*-deficient mice, whereas osteophyte formation at the tibial compartment did not differ (Fig. 5E). Histological scoring of osteophytes in the same mice showed reduced osteophyte formation in mice lacking *Cxcl9* at 4 weeks post-operatively (Fig. 5F). At 8 weeks after ACLT, micro-computed osteophyte evaluation confirmed these findings and demonstrated significantly reduced osteophyte volume in *Cxcl9*-deficient mice on the femoral site and the total joint, while tibial osteophyte formation did not show any differences between the groups (Fig. 5G). Histological assessment of osteophytes at this time point further revealed a decreased femoral osteophyte formation in *Cxcl9*-deficient mice (Fig. 5H, **Supplementary Fig. 2**). Overall, in early and advanced OA, *Cxcl9* seems to protect from osteophyte formation, particularly in the femoral compartment.

### Reduced synovitis in OA knees upon ablation of Cxcl9

**Fig. 6:**
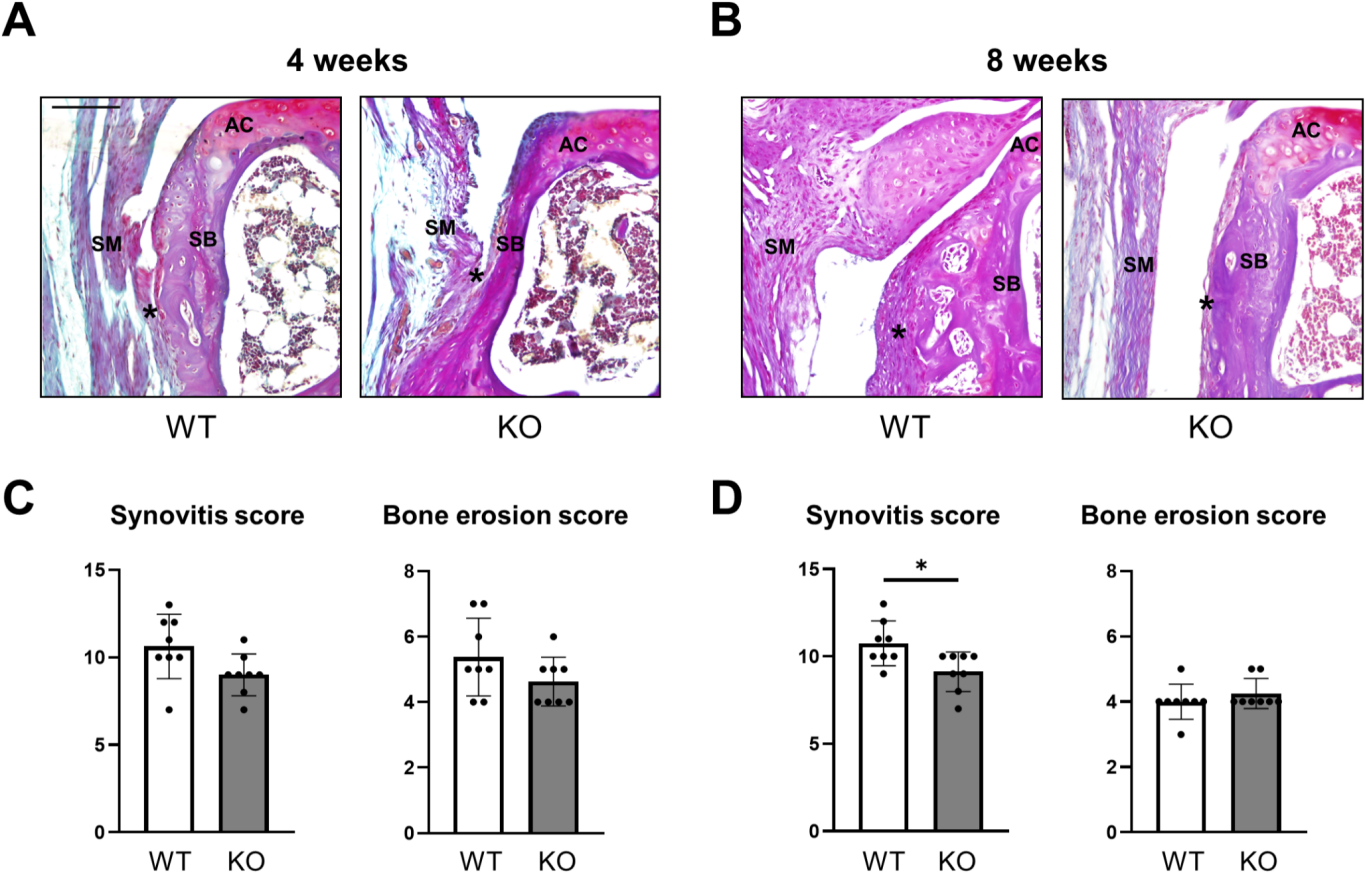
Cxcl9-deficiency reduces synovitis after ACLT. **A** Representative BIC-stained images of the tibial knee joint for synovium and bone erosion 4 weeks after ACLT (scale bar = 100 μm). Asterisks indicate synovial hyperplasia and pannus formation with bone erosion. AC, articular cartilage; SB, subchondral bone; SM, synovium. **B** Representative BIC-stained images of the tibial knee joint for synovium and bone erosion 8 weeks after ACLT (scale bar = 100 μm). **C** Histological synovitis scoring and bone erosion scoring of total joints 4 weeks after ACLT. **D** Histological synovitis scoring and bone erosion scoring of total joints 8 weeks after ACLT. The data are expressed as the means ± SD, *n* = 8 per group as indicated. Two-tailed Student’s t-test was used for data analysis. ^*^P < 0.05 compared as denoted by bar

Evaluation of synovial inflammation and adjacent bone erosion, two hallmark manifestations of OA, was performed on BIC-stained sections of ACLT knees (Fig. 6A, B). Histological scoring 4 weeks after ACLT revealed that synovitis and bone erosion were alleviated only by tendency in *Cxcl9*-deficient mice (Fig. 6C). At 8 weeks after ACLT, synovial inflammation was significantly decreased in *Cxcl9*-deficient mice, while bone erosion did not differ between *Cxcl9*-deficient and WT mice (Fig. 6D). Therefore, *Cxcl9* seems to modify the inflammatory responses in the synovial membrane during the progression of experimental OA.

### The expression of interleukin-1beta is decreased while cartilage markers are overexpressed in OA knees from Cxcl9-deficient mice

**Fig. 7:**
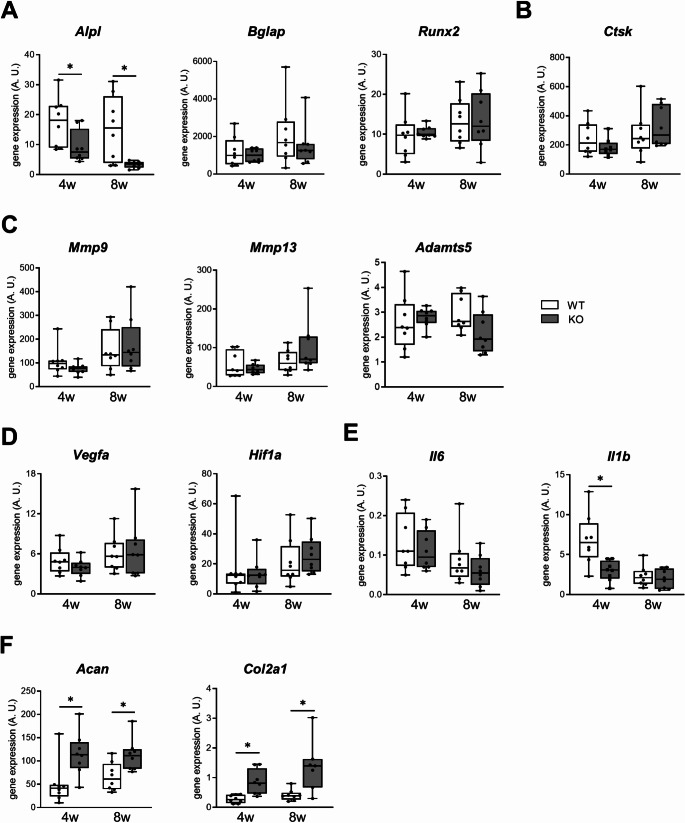
The gene expression of inflammatory and cartilage markers are affected in OA knees from Cxcl9-deficient mice. **A-F** qRT-PCR expression analysis for the indicated genes in OA knees of WT and *Cxcl9*-deficient mice at 4 weeks and 8 weeks after ACLT. *Alpl* = alkaline phosphatase; *Bglap* = bone gla-protein; *Runx2* = runt-related transcription factor 2; *Ctsk* = cathepsin k; *Mmp9* = matrix metalloproteinase 9; *Mmp13* = matrix metalloproteinase 13; *Adamts5* = a disintegrin and metalloproteinase with thrombospondin motifs 5; *Vegfa* = vascular endothelial growth factor a; *Hif1a* = hypoxia-inducible-factor-1 alpha; *Il1b* = interleukin-1 beta; *Il6* = interleukin-6; *Acan* = aggrecan; *Col2a1* = collagen type 2 alpha 1. The data are presented as median with minimum and maximum values (whiskers), *n* = 8 per group as indicated. Mann-Whitney-U-test was used for data analysis. ^*^P < 0.05 compared as denoted by bar

To validate our findings on a molecular level, we finally monitored gene expression in ACLT and contralateral healthy knees 4 and 8 weeks after surgery in an independent set of WT and *Cxcl9*-deficient mice. Here, the osteoblast marker *Alpl* (Alkaline phosphatase) was significantly decreased in OA knees of Cxcl9-deficient mice at both early and late time points, while no changes in the expression of the *Bglap* (bone gla-protein) and *Runx2* (runt-related protein 2) (Fig. 7A), the osteoclast markers *Ctsk* (cathepsin K) (Fig. 7B), the cartilage catabolic markers *Mmp9* (matrix metalloproteinase 9), *Mmp13*, and *Adamts5 (A disintegrin and metalloproteinase with thrombospondin motifs 5)* (Fig. 7C), or the key regulator of angiogenesis, *Vegfa* (vascular endothelial growth factor a) and *Hif1a* (hypoxia-inducible factor 1a) (Fig. 7D), between *Cxcl9*-deficient and WT mice were observed. However, while *Il6* (interleukin-6) was unaltered, a reduced expression of *Il1b* (interleukin-1beta), a master pro-inflammatory cytokine in joint inflammation, was significantly reduced in the OA knees of *Cxcl9-deficient* mice 4 weeks after ACLT (Fig. 7E). Moreover, the chondrocyte markers *Acan* (aggrecan) and *Col2a1* (collagen type 2 alpha 1) were significantly increased in OA knees of *Cxcl9*-deficient mice at both early and late time points (Fig. 7F). Together, these results provided further molecular evidence that *Cxcl9*-deficiency benefits cartilage marker expression and reduces induction of pro-inflammatory stimuli.

## Discussion

Based on its pleiotropic functions in bone remodeling and inflammation, and its increased levels in the knees of patients with joint degeneration, the present study was designed to test a pathophysiological role of CXCL9 in experimental OA. Using the highly standardized ACLT model, which mimics post-traumatic OA, we found that genetic ablation of CXCL9 protected against cartilage degeneration, osteophyte formation, and synovitis. However, there was no alleviation of pathological changes in the subchondral bone compartment in mutant mice. Therefore, the data suggest that the chemokine CXCL9 primarily affects articular cartilage integrity and joint inflammation, while not interfering with pathological processes in subchondral bone remodeling.

From a clinical perspective, elevated levels of CXCL9 in synovial fluid and peripheral blood samples have previously been described in patients with OA [14]. In a different study, CXCL9 levels in the peripheral blood and bone marrow of healthy individuals increased with age [26], suggesting that multiple factors may alter local and systemic CXCL9 levels. Using a more practical approach, Nees et al. described that knee function was correlated inversely with synovial CXCL9 concentrations in patients with OA undergoing arthroplasty surgery [13]. Even though these results do not provide implicit evidence for a causal link between CXCL9 and OA progression, they pointed towards a potential role of this chemokine in degenerative joint disease.

In this regard, our experimental findings support an important role of CXCL9 in mediating specific pathophysiological aspects relevant for OA progression. Consistent with clinical reports, we found increased expression of *Cxcl9* and its receptor *Cxcr3* in whole joints of ACLT mice. Comparing cartilage degeneration using OARSI scoring, we found that *Cxcl9*-deficient mice showed improved cartilage integrity at 4 and especially 8 weeks after ACLT. Moreover, cartilage calcification and synovitis were less pronounced in *Cxcl9*-deficient mice with late-stage OA compared to WT controls, and mutant mice showed a strong attenuation of osteophyte formation during OA progression. Although we cannot rule out a direct negative effect of CXCL9 on chondrocytes, these findings are suggestive of an overall immunomodulatory impact of CXCL9 in these disease manifestations.

While these observations indicate a protective effect of *Cxcl9*-deficiency on joint integrity during experimental OA, the pathological remodeling of subchondral bone, another hallmark of OA progression, was not affect in mutant mice. This is indeed surprising, as CXCL9 was previously reported to regulate recruitment of osteoclast precursors in fish [10] and to inhibit bone formation in the murine skeleton. For example, it has been demonstrated that blockage of CXCL9 via neutralizing antibodies prevented bone loss in WT mice with experimental osteoporosis [11]. In addition, Huang et al. demonstrated that *Cxcl9* expression in osteoblasts is regulated downstream of mammalian target of rapamycin complex 1 (mTORC1) and signal transducer and activator of transcription 1 (STAT1) signaling. Once secreted, CXCL9 was shown to bind to VEGFA, thus inhibiting bone angiogenesis and osteogenesis [27]. In this regard, the progression of OA is associated with angiogenesis in the synovium, cartilage and in osteophytes [28]. Furthermore, increased vascularization can disrupt the osteochondral junction and increase joint destruction [29]. Surprisingly, *Cxcl9*-deficient mice did not show an exacerbated course of OA, with unaltered subchondral bone architecture and osteoclast parameters. With respect to clinically relevant outcome measures, *Cxcl9*-deficient mice were even protected from cartilage degeneration and synovitis. Thus, our findings indicate that the pro-inflammatory role of CXCL9, rather than its angiostatic properties or the modulation of bone resorption and bone formation, is of pathophysiologic relevance in experimental OA. This notion is further supported by the results of gene expression analysis of OA knee joints in *Cxcl9*-deficient and WT mice at early and late stages of disease progression. Consistent with the radiological and histological findings in the subchondral bone compartment, the expression of osteoblast and osteoclast marker genes, except for *Alpl*, was not different between mutant and control mice in experimental OA at both time points. In this regard, the observed decrease in *Alpl* expression may reflect the decreased osteophyte formation in *Cxcl9*-deficient mice. Regarding the role of CXCL9 in angiogenesis, we observed unaltered expression of the key angiogenetic factors *Vegfa* and *Hif1a*. Although we did not specifically study angiogenesis during OA progression, these results make it unlikely that the proposed role in skeletal vascularization is predominant in the pathophysiological impact of CXCL9 in experimental OA. In sharp contrast, the expression of key cartilage markers was significantly increased in *Cxcl9*-deficient mice both at early and late stages of OA progression. Mutant mice showed higher levels of *Acan* mRNA, which encodes aggrecan with essential function for elasticity in hyaline cartilage. Consistent with this, higher expression of *Col2a1*, one of the most abundant components in articular cartilage, was found in *Cxcl9*-deficient mice 4 and 8 weeks after ACLT. These data essentially support the results of the histomorphometric analysis, which showed an increased ratio of hyaline to calcified cartilage in the *Cxcl9*-deficient group. Finally, analysis of pro-inflammatory cytokines revealed no significant changes in *Il6*, however, we observed a pronounced reduction of *Il1b* expression in OA knees in mutant mice 4 weeks after ACLT. Together with the reduced synovitis scores observed histologically, these combined results suggest that CXCL9 may influence the progression of OA by modulating at least some aspects of the vast array of pro-inflammatory responses in degenerative joint disease.

This study has several limitations. First, we used only female mice because women have a higher risk of developing OA than men and to provide optimal comparability with other OA reference studies. Nevertheless, hormonal factors could potentially affect outcome measurements, and different results may be obtained when subjecting male *Cxcl9*-deficient mice to ACLT. Furthermore, a global deficiency model was employed in this work. Even though *Cxcl9*-deficient mice display normal skeletal phenotype, we cannot fully exclude whether CXCL9 might affect development and joint structures prior to ACLT surgery, and thus the changes in response to ACLT may be related to developmental changes occurring over time. In addition, the present results do not allow the differentiation between cells which are expressing Cxcl9 and Cxcr3 in the joint. Hence, further studies employing conditional *Cxcl9*-deficiency models are warranted to delineate the precise cellular and molecular mechanisms by which CXCL9 affects joint integrity during experimental OA. Finally, the ACLT model, which was used to induce post-traumatic OA in our study, is a well-established model for experimental OA and has been employed in a multitude of previous studies [17, 29–31]. However, it should be acknowledged that ACLT commonly yields a more severe OA phenotype than other models, e.g., the destabilization of the medial meniscus or pathological weight bearing and degenerative processes due to ageing without surgical intervention [16, 32].

## Conclusions

Our results demonstrate that genetic inactivation *Cxcl9* leads to an attenuated course of experimental OA and protects from cartilage erosions and calcification as well as synovial inflammation. In contrast, the pathological remodeling of subchondral bone is not affected in *Cxcl9*-deficient mice. Although we cannot rule out a direct negative impact on chondrocytes, our combined results point towards a pro-inflammatory effect of CXCL9 in the progression of OA. Therefore, pharmacological antagonization of CXCL9-signalling could represent a novel approach to attenuate the severity of post-traumatic OA in patients and to reduce the number of required arthroplasties including their complications.

## Electronic supplementary material

Below is the link to the electronic supplementary material.

Supplementary Material 1

## Data Availability

No datasets were generated or analysed during the current study.
