# Supplementary material for: Cxcl9-deficiency attenuates the progression of post-traumatic osteoarthritis in mice
Source: Inflamm Res. 2025 Mar 6;74(1):48. doi: 10.1007/s00011-025-02013-8 (PMC11885341; doi:10.1007/s00011-025-02013-8)
Supplement: Supplementary file 1 — Supplementary Material 1 [file 11_2025_2013_MOESM1_ESM.docx]

**Supplementary material**

***Cxcl9*-deficiency attenuates the progression of post-traumatic osteoarthritis in mice**

Antonia Donat^1†^, Weixin Xie^1†^, Shan Jiang^1^, Laura Janina Brylka^2^, Thorsten Schinke^2^, Tim Rolvien^1^, Karl-Heinz Frosch^1,3^, Anke Baranowsky^1,*^, and Johannes Keller^1,*^

^1^Department of Trauma and Orthopedic Surgery, University Medical Center Hamburg-Eppendorf, 20251 Hamburg, Germany.

^2^Department of Osteology and Biomechanics, University Medical Center Hamburg-Eppendorf, 20251 Hamburg, Germany.

^3^Department of Trauma Surgery, Orthopedics and Sports Traumatology, BG Hospital Hamburg, 21033 Hamburg, Germany.

† These authors contributed equally to this work.

* Co-supervised the study

Correspondence: Johannes Keller; Department of Trauma and Orthopedic Surgery, University Medical Center Hamburg-Eppendorf; Martinistraße 52, 20251 Hamburg; Germany; Telephone: +49 (0)40 7410 5 6691; Fax: +49 (0)40 7410 4 0085; Email: [j.keller@uke.de](mailto:j.keller@uke.de)


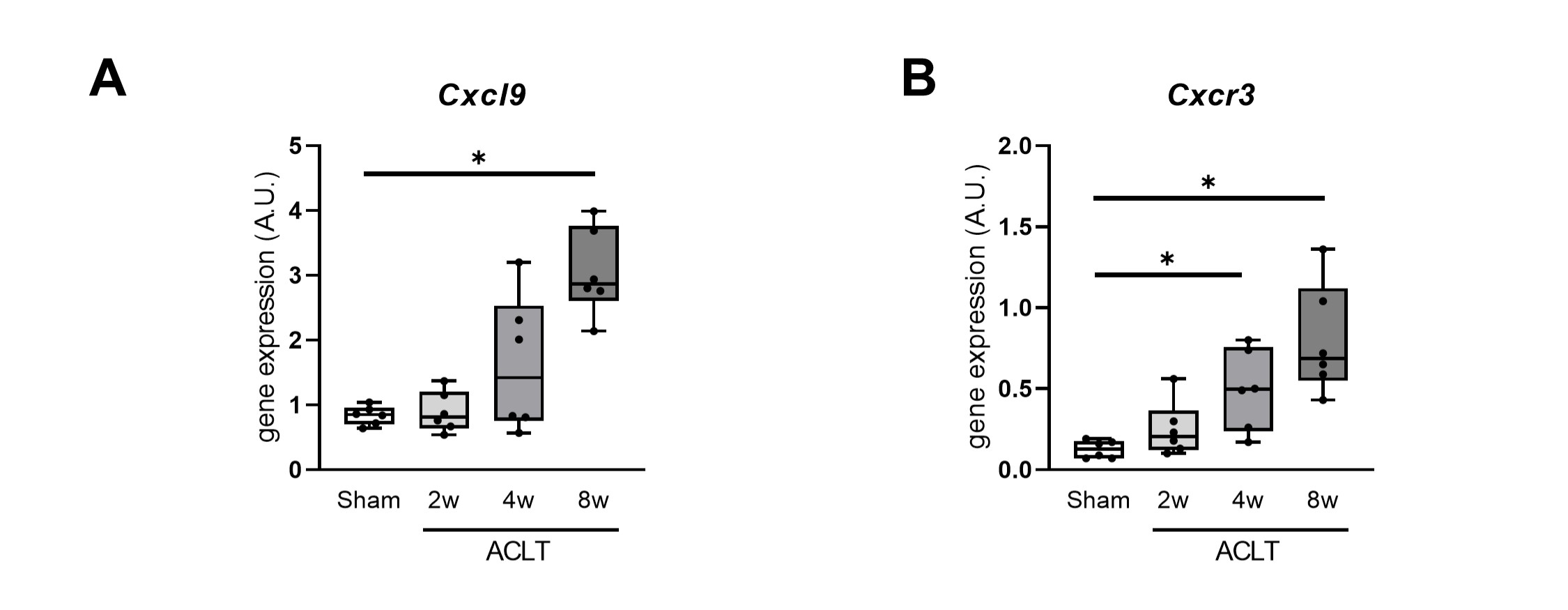


**Fig. S1.** **Increased gene expressions of Cxcl9 and Cxcr3 in knee joints during OA progression.** (**A**) qRT-PCR expression analysis for the *Cxcl9* gene in WT sham or OA knees at indicated time points. (**B**) qRT-PCR expression analysis for the *Cxcr3* gene in WT sham or OA knees at indicated time points. The data are presented as median with minimum and maximum values (whiskers), n = 6 per group as indicated. One-way ANOVA followed by Tukey’s post-hoc test was used for data analysis. ^*^*P* < 0.05 compared as denoted by bar.


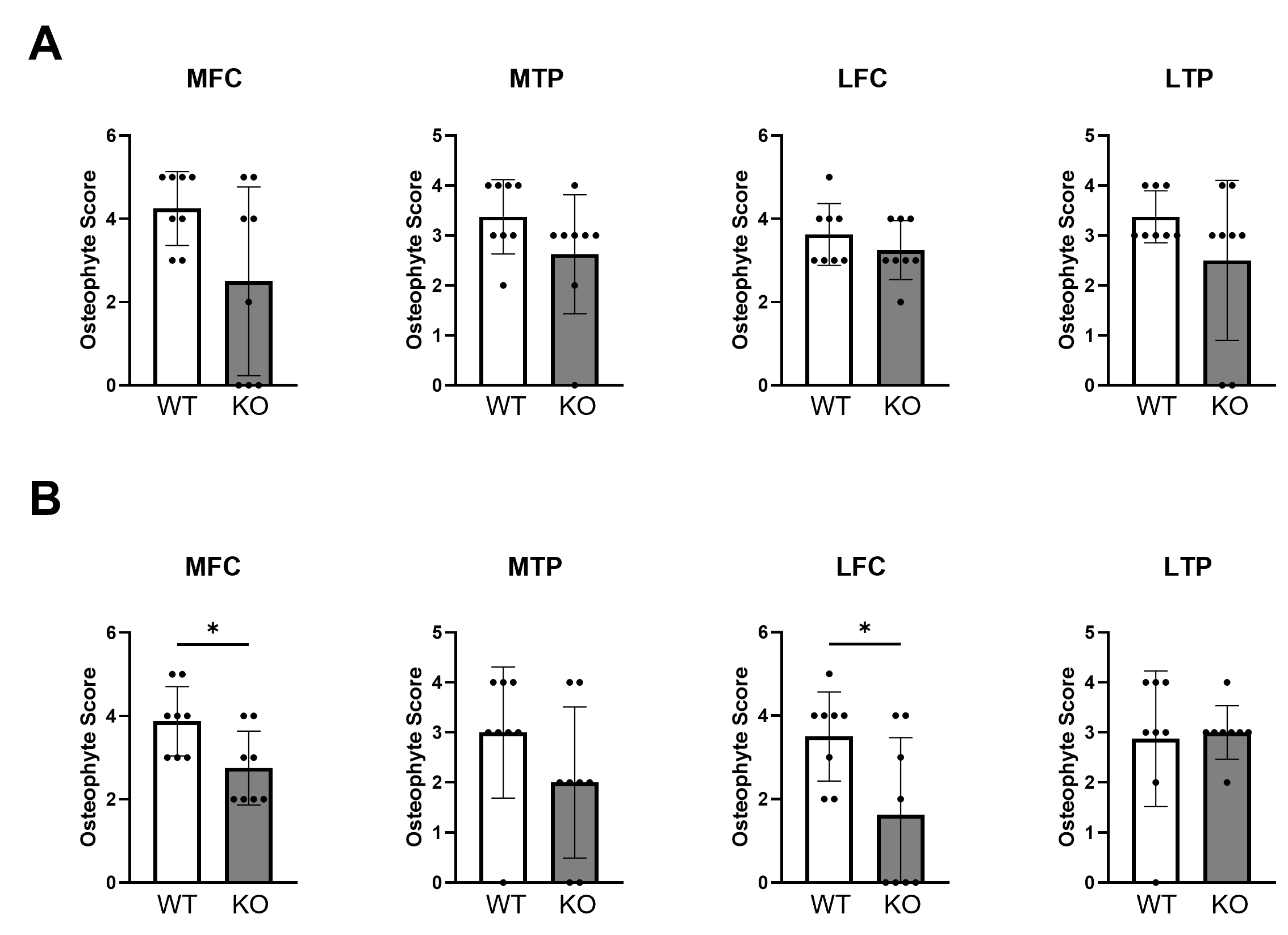


**Fig. S2.** **Supplementary data related to Fig. 5.** (**A**) Histological osteophyte scoring of medial femoral condyle (MFC), medial tibial plateau (MTP), lateral femoral condyle (LFC), and lateral tibial plateau (LTP) at 4 weeks after ACLT. (**B**) Histological osteophyte scoring of MFC, MTP, LFC, and LTP at 8 weeks after ACLT. The data are expressed as the means ± SD, n = 8 per group as indicated. Two-tailed Student’s t-test was used for data analysis. ^*^*P* < 0.05 compared as denoted by bar.
